# A relatively high zoonotic trematode prevalence in *Orientogalba ollula* and the developmental characteristics of isolated trematodes by experimental infection in the animal model

**DOI:** 10.1186/s40249-022-01014-7

**Published:** 2022-08-19

**Authors:** Jian Li, Yijing Ren, Lei Yang, Jiani Guo, Haiying Chen, Jiani Liu, Haoqiang Tian, Qingan Zhou, Weiyi Huang, Wei Hu, Xinyu Feng

**Affiliations:** 1 Basic Medical College, Guangxi Traditional Chinese Medical University, Nanning, 530005 Guangxi China; 2 College of Life Sciences, Inner Mongolia University, Hohhot, 010070 China; 3 College of Animal Science and Technology, Guangxi University, Nanning, 530005 Guangxi China; 4 National Institute of Parasitic Diseases, Chinese Center for Disease Control and Prevention, Key Laboratory of Parasite and Vector Biology of China Ministry of Health, WHO Collaborating Centre for Tropical Diseases, Joint Research Laboratory of Genetics and Ecology On Parasite–Host Interaction, Chinese Center for Disease Control and Prevention, Fudan University, 200025 Shanghai, China; 5 Department of Infectious Diseases, Huashan Hospital, State Key Laboratory of Genetic Engineering, Ministry of Education Key Laboratory for Biodiversity Science and Ecological Engineering, Ministry of Education Key Laboratory of Contemporary Anthropology, School of Life Sciences, Fudan University, Shanghai, 200438 China; 6 School of Global Health, Chinese Center for Tropical Diseases Research, Shanghai Jiao Tong University School of Medicine, Shanghai, 20025 China; 7 One Health Center, Shanghai Jiao Tong University-The University of Edinburgh, Shanghai, 20025 China

**Keywords:** *Orientogalba ollula*, Zoonotic trematode, Intermediate host, Prevalence, ITS2

## Abstract

**Background:**

Food-borne parasitic diseases decrease food safety and threaten public health. The snail species is an intermediate host for numerous human parasitic trematodes. *Orientogalba ollula* has been reported as intermediate hosts of many zoonotic trematodes. Here, we investigated the prevalence of zoonotic trematodes within *O. ollula* in Guangxi, China, and assessed their zoonotic potential.

**Methods:**

Snails were collected from 54 sites in 9 cities throughout Guangxi. The snail and trematode larvae species were determined by combining morphological characteristics and molecular markers. The trematodes prevalence and constituent ratio were calculated and compared among different habitat environments. Phylogenetic trees of the trematode species were constructed using the neighbor-joining method with nuclear internal transcribed spacer 2 (ITS2) sequences. The developmental cycles of the isolated trematodes were examined by experimental infection in ducks. The developmental characteristics of *Echinostoma revolutum* was recorded by dissecting infected ducklings from 1-day post infection (dpi) to 10 dpi.

**Results:**

The overall prevalence of trematode larvae was 22.1% (1818/8238) in *O. ollula* from 11 sample sites. Morphological together with molecular identification, showed that *E. revolutum*, *Australapatemon* sp., *Hypoderaeum conoideum*, *Pharyngostomum cordatum*, and *Echinostoma* sp. parasitized *O. ollula,* with the highest infection rate of *E. revolutum* (13.0%). However, no *Fasciola* larvae were detected. The trematodes prevalence and constituent ratio varied in two sub-biotypes (*P* < 0.01). A neighbor-joining tree analysis of ITS2 sequences resulted in distinct monophyletic clades supported by sequences from isolated larvae with high bootstrap values. Ducklings exposed to *O. ollula* infected with *Echinostoma* sp., *E. revolutum*, and *H. conoideum* larvae were successfully infected. The animal model for *Echinostoma revolutum* was successfully established. *E. revolutum* matured from larvae to adult at 10 dpi in the intestine of the duck, and the developmental characteristics of *E. revolutum* were characterized by the maturation of the reproductive and digestive organs at 6–8 dpi.

**Conclusions:**

This study revealed a high prevalence of zoonotic trematodes in *O. ollula* from Guangxi, China. Existing trematodes infection in animals and human clinical cases, coupled with the wide geographical distribution of *O. ollula*, necessitate further evaluations of the potential risk of spillover of zoonotic infection from animal to human and vice versa.

**Graphical Abstract:**

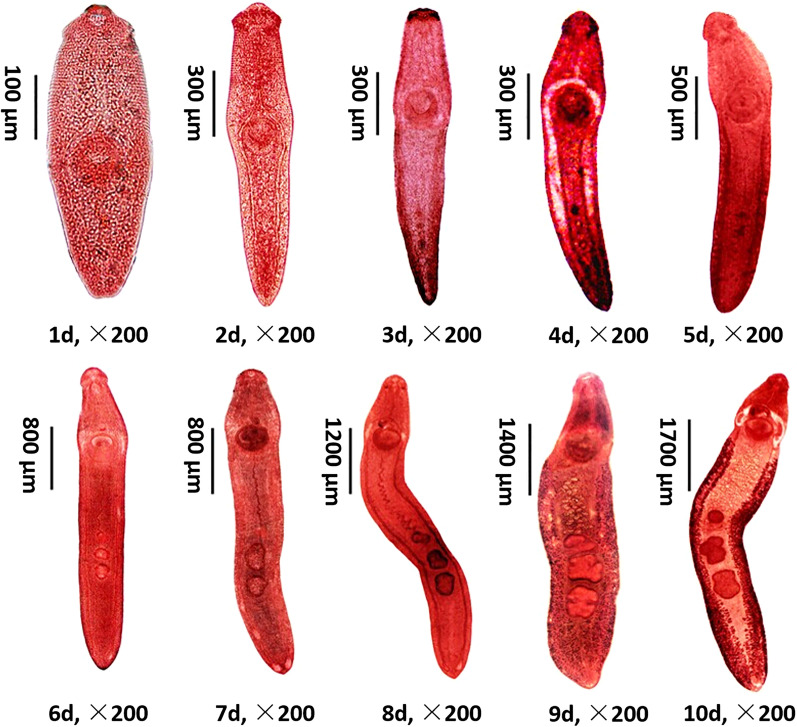

**Supplementary Information:**

The online version contains supplementary material available at 10.1186/s40249-022-01014-7.

## Background

Food-borne trematodes can infect many mammals, including livestock and humans, and cause severe veterinary and public health problems [1, 2]. In addition to clonorchiasis**,** other common food-borne fluke diseases include fascioliasis, paragonimiasis, and echinostomiasis. Corresponding trematodes belong to the families Fasciolidae, Paragonimidae and Echinostomatidae [3–5]. Although human infection is opportunistic, several clinical cases have recently been reported [6–8]. Notably, multi-infection of *Clonorchis sinensis*, Heterophyidae, and Echinostomatidae was reported in China [2]. Additionally, in Northern Vietnam, a high prevalence of zoonotic trematodes was identified in more than half of the fish species investigated [9]. However, knowledge gaps in the taxonomic impediment*,* pathology, and epidemiology may underestimate the disease burden on humans, livestock, and wild animals.

Typically, an intermediate snail host may play a vital role in the transmission of these zoonotic diseases [10]. Previous studies revealed that *Orientogalba ollula* (Gastropoda: Lymnaeidae) is an intermediate host for a variety of trematodes such as *Fasciola gigantica*, *F. hepatica*, *Echinostoma revolutum*, and *Echinochasmus perfoliatus* [11]. Morphologically, the shell of *O. ollula* is thin and translucent with an ear-shaped aperture, and the apex/body ratio is 1.25 (10/8 mm). From an ecological perspective, oviparous hermaphroditic *O. ollula* usually lives in large aggregation in suitable environments, for example, sewage sludge bottoms or among broken bricks, and feeds on algae, humus, and aquatic plants, and its natural habitats include lakes, canals, ponds and rice fields [12]. *O. ollula* is widely distributed in China, Japan, and other Asian countries, and was introduced from Southeast Asia into Australia [13]. During the past decade, many efforts have been made to prevent the spread of the invasive snail. However, the interrelationships between invasive snail species and epidemiological roles in zoonotic disease have not been extensively explored [11, 14].

Zoonotic trematode infections can result from eating raw or undercooked aquatic products such as vegetables and freshwater fish. Raw vegetables, for instance, the most common lettuce (*Lactuca sativa*), watercress (*Nasturtium officinale*), parsley (*Petroselinum crispum*), and fish mint (*Houttuynia cordata*), may get contaminated by trematode larvae attached to them [15]. From 2011 to 2012, a series of human *F. gigantica* infection cases were confirmed in Binchuan County, Dali Prefecture, Yunnan Province, China [7]. Epidemiological investigation presumed that fish mint consumption was most likely the cause of the infections, and the trematode shed from the snail hosts was the causative agent. In fact, similar cases related to the practice of dining on raw vegetables have also occurred in Myanmar, Thailand, North-east Ethiopia, and other countries [9, 16, 17]. Guangxi Zhuang Autonomous Region is contiguous to Yunnan Province and has similar climate conditions. Importantly, most local residents in the two regions share similar lifestyles and dietary habits. Given that *O. ollula* has a wide distribution in Guangxi, it is not only an important intermediate host of *Fasciola* [10, 18], but also may act as a potential reservoir for other parasitic zoonoses. Therefore, the main objective of this study was to identify the prevalence of various trematode larvae in *O. ollula*, and to assess the zoonotic potential of trematodes for public health awareness in Guangxi.

## Methods

### Study areas and snail collection

To investigate the potential vector capacity of *O. ollula* in Guangxi, snails were collected from 54 sites in 9 cities, namely Beihai, Fangchenggang, Guigang, Guilin, Liuzhou, Laibin, Nanning, Qinzhou, Wuzhou, and Yulin, from 2012 March to 2020 October (the number of snail samples per site was about 200). Details of each locality sampled are given in Additional file 2: Table S1. At each sampling site, the snails were collected manually by a plastic scoop, transported to the laboratory, cleaned, rinsed five times in sterilized water, and then placed in plastic trays for subsequent experiments.

### Identification of snails and isolation of trematodes larvae

Preliminary snail identification was based on morphological characters using standardized taxonomic keys as described by Liu [12]. Meanwhile, the snail identifications were confirmed by the internal transcribed spacer 1 (ITS1) sequence as described by Correa et al. [17] using at least 20 samples randomly selected from one site. The foot and soft body tissue of identified *O. ollula* were necropsied for trematode larvae (rediae, cercariae, or metacercariae). The body length and body width of rediae and the body length, body width, tail length, and tail width of cercariae at each site were captured by a Motic BA400 microscope (Motic, Xiamen, China) and measured under a micrometer. The diameter and wall thickness of metacercaria were also recorded.

### Molecular examinations of the trematodes

A single larva with the identical morphology at each sampling site was selected and rinsed with sterilized distilled water three times before being used to extract parasite genomic DNA by a DNeasy Blood & Tissue kit (Qiagen, Hilden, Germany) according to manufacturer instructions. We selected at least 20 parasite larvae of different developmental stages for molecular identification. The extracted DNA samples were stored at − 20 ℃ until PCR amplification. A PCR assay targeting the sequence of the internal transcribed spacer 2 (ITS2) gene was used to amplify the desired amplicons. The universal primer pairs were designed as McManus et al. described [18]. The sequences of the primers are listed as: 3S-F (5′-GGTACCGGTGGATCACTCGGCTCGTG-3′) and A28-R (5′-GGGATCCTGGTTAGTTTCTTTTCCTCCGC-3′). All the PCR products were directly sequenced after being purified. The obtained sequences were edited using DNASTAR software (www.dnastar.com/software/lasergene/) and aligned using ClustalX (http://www.clustal.org/clustal2/). The identity of individual specimens was ascertained by comparison with the sequences available in the 'non-redundant' database in GenBank by BLAST (http://www.ncbi.nlm.nih.gov/blast/). The nucleotide sequences obtained in the present study have been deposited in the GenBank database under the accession numbers.

### Phylogenetic tree construction with ITS2

Briefly, phylogenetic trees were inferred using the neighbor-joining (NJ) method in MEGAX (26). Molecular phylogenetic trees were constructed using our sequencing results of *Echinostoma* sp., *E. revolutum* and *H. conoideum* and the closely related isolates retrieved from NCBI GenBank, including *E. robustum* (LC224084), *E. friedi* (AJ564383), *E. miyagawai* (MW199188), *E. paraensei* (AF336232), *E. caproni* (AJ564382), *E. trivolvis* (GQ463127), and *E. malayanum* (JF412727). *F. gigantica* (MK321643), isolated from a cow, was used as an out-group. Similarly, the NJ phylogenetic tree of *Australapatemon* sp. was constructed after alignments with multiple sequence retrieved from NCBI GenBank, including *Pharyngostomum cordatum* (OL870492 and KJ137231), *A. burti* (KU950451), *Austrodiplostomum ostrowskiae* (KT72878), *Alaria americana* (MH521246), *Diplostomum paracaudum* (KJ889013) and *Cyathocotyle prussica* (MH521249), *Brachylaima* sp. (JX010634), and *Schistosoma japonicum* (S72866) used as out-group.

### Prevalence of trematodes in *O. ollula*

The overall trematode prevalence was calculated as the number of snails infected with the parasite divided by the total number of snails examined. The prevalence of trematode infection with a given parasite species was also calculated. Based on the ecological environment of the collection site, two types of areas were included: Type 1 areas were rice cultivation areas (51 sites, marked by circular shapes in Additional file 1: Fig. S1); Type 2 areas were the vegetation areas of crops, which were often used as raw food (10 sites, marked by triangular shape in Additional file 1: Fig. S1). We further compared the trematodes constituent ratio among the subset of sites using the Chi-squared tests.

### Experimental infections and developmental characteristics of isolated trematodes in the intestine of duck

Six 5-day-old ducklings were fed with snails parasitized by isolated trematodes in the field. All the isolated trematodes were first used to establish an animal infection model. We treated ducklings by oral gavage with isolated metacercariae in physiological saline solution. However, this approach failed to get duckling infected after observation, so we altered the procedure to feed each duckling with 20 *O. ollula* (each snail was crushed and examined microscopically as infected snails by the presence of metacercariae), and one duckling was dissected every day from the 1st to the 10th day after ingestion. The trematodes were collected from the duck intestines using an improved method of helminthological dissection [19], and high-resolution pictures of the collected trematodes were taken with a Motic BA400 microscope and additional accessories. Carmine staining of the fixed specimen was made according to the method described by Fanyao [20], and collar, spines, oral sucker, acetabulum, prepharynx, esophagus, testis, and ovary were measured from digital images made during daily observations. After repeated rinsing with sterilized distilled water for 2–3 times, DNA extraction was carried out using the above method. The ITS2 gene was amplified and sequenced using the same method, and the trematode species was verified.

## Results

### Overall information on the sampling and survey data

**Fig. 1:**
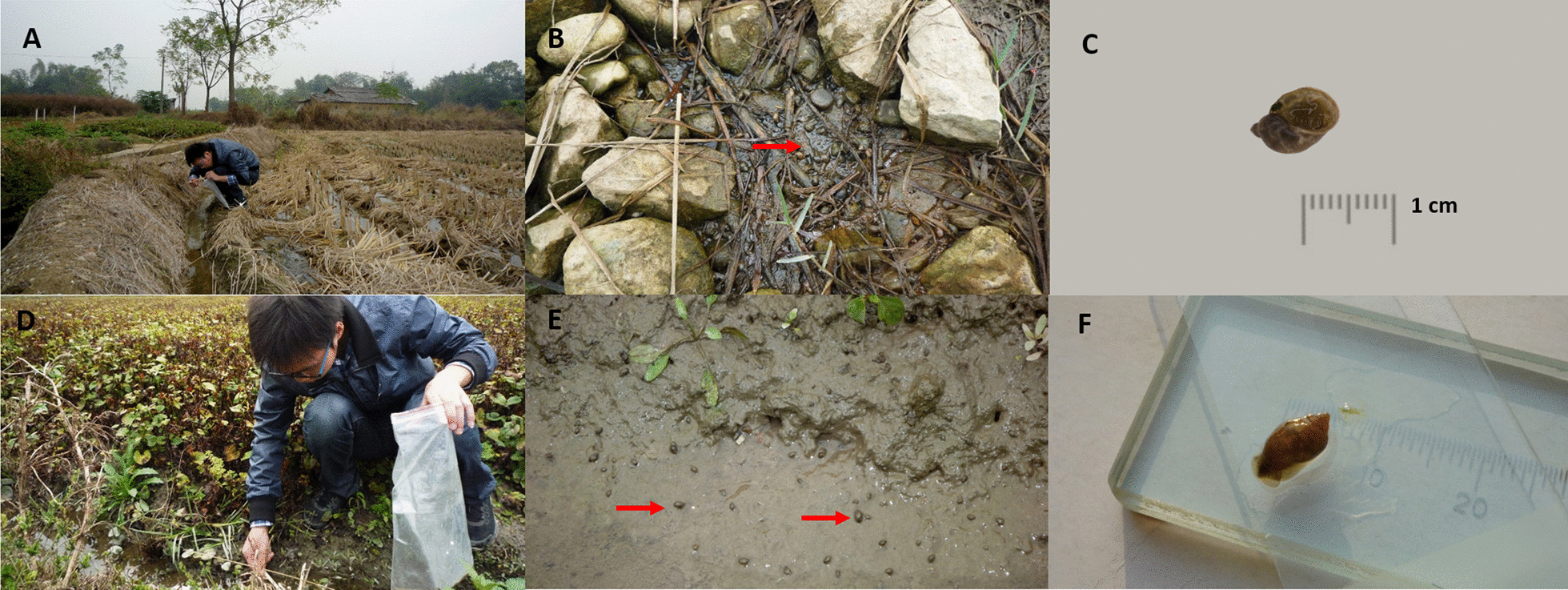
Detailed information related to the sampling sites, sample selection, and method of collecting information. **A**, **B** Typical ecological environment of *O. ollula* (Type 1 areas: rice cultivation areas); **C** *O. ollula* image in anterior view; **D**, **E** Typical ecological environment of *O. ollula* (Type 2 areas: vegetation areas of agricultural crops which often used as the raw food); **F** *O. ollula* image posterior view

Snail samples of *O. ollula* were collected from 54 sites (shown in Fig. 1 and Additional file 1: Fig. S1), with 38–214 snails from each site. Trematodes were found in 17 sites investigated following dissection, including Tianbao Reservoir and Hede village in Nanning city; Liushan Town, Liutang Village, Guangrong village, and Cha Village in Liuzhou City; and Maling Town in Guilin City. During the dissections, different developmental stages of the trematode (redia, cercaria and metacercariae) were found.

### Species identification of *O. ollula*

Morphological features of *O. ollula* were identified by traditional quantitative shell characteristics (shell: medium-sized to large, thin, colour brown or yellowish; shell height: 10 mm; shell width: 8 mm; four up to five separate brown spiral bands; aperture oval, slit-like umbilical foramen). *O. ollula* collected in this study were small, oval, and had a thin shell and a small aperture (Fig. 1C, F). The ITS1 data of all sequenced snails were 100% consistent with those of the previously reported sequences in GenBank under the accession number HM769888–HM769898.

### Morphological character descriptions and molecular identification of trematode larvae

**Fig. 2:**
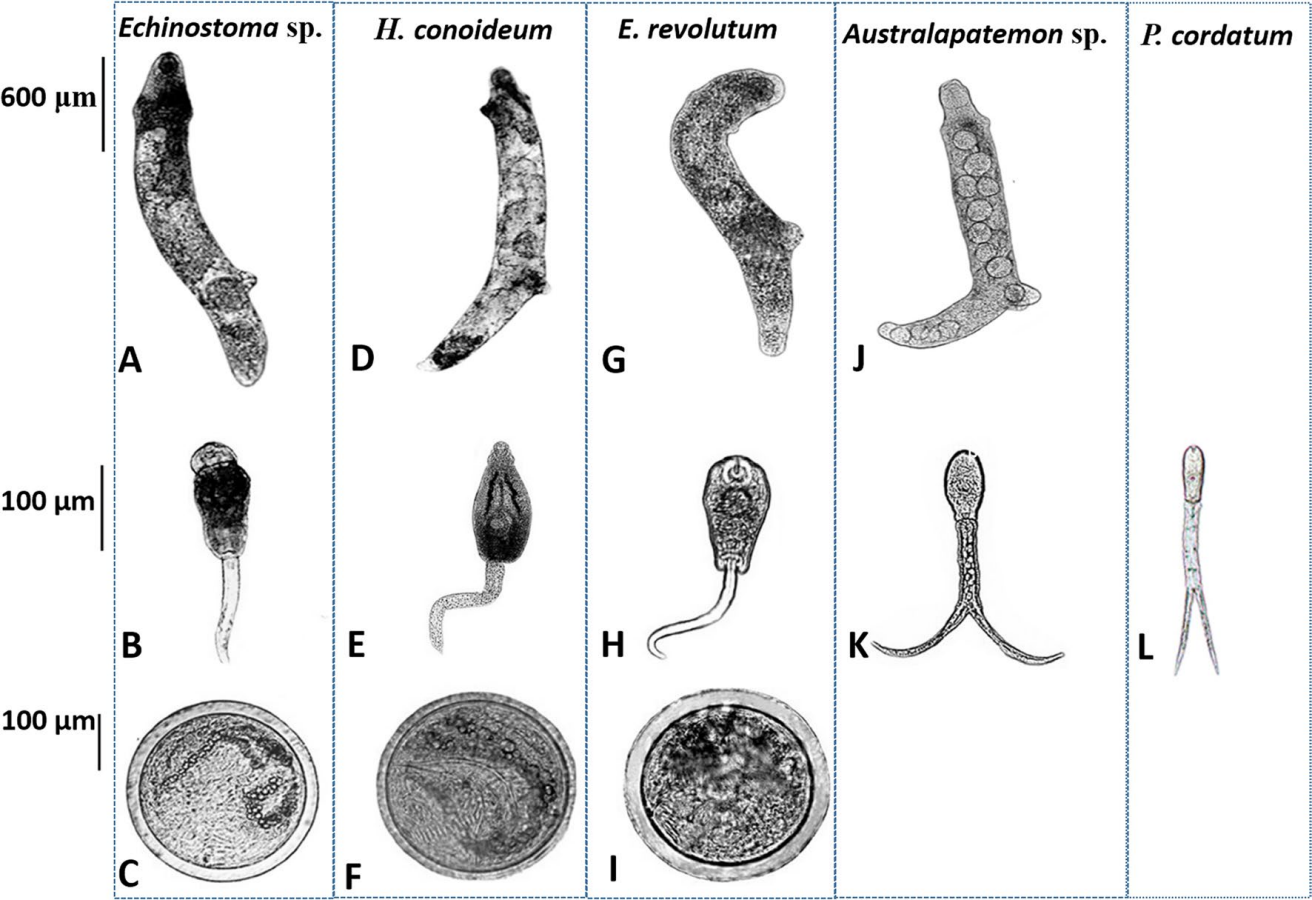
Morphology of rediae, cercariae, and metacercariae collected in *O. ollula*. **A**–**C** Rediae, cercariae and metacercariae of *Echinostoma* sp.; **D**–**F** Rediae, cercariae and metacercariae of *Hypoderaeum conoideum*; **G**–**I** Rediae, cercariae and metacercariae of *Echinostoma revolutum*; **J**, **K** Rediae and cercariae of *Australapatemon* sp.; **L** Cercariae of *Pharyngostomum cordatum*

Generally, morphological characters of three echinostomes (*Echinostoma* sp., *H. conoideum*, and *E. revolutum*) showed considerable morphological similarity (Fig. 2). The rediae were cylindrical, blunt at both ends, slightly pointed at the head, and more pointed at the tail. The body was curved to the ventral surface with muscular feet, and the movement was slow. The tail of the cercariae was not forked (Fig. 2B–H). Beyond the general similarities, the head of *H. conoideum* cercariae showed prominent spines, as well as well-developed ventral suckers, pharynx, and intestines (Fig. 2D–F). The metacercariae were round and had two transparent walls, and the outer wall was thicker than the inner wall. Abdominal suckers and refractive granules of larvae could be seen inside the cyst. Due to the movement of the larvae inside the sac, the small spines around its head were not easily observed. The rediae of *Australapatemon* sp. forms a distinct bulge at the head. The cercaria larvae had a forked tail, which was longer than the body length. The cercaria of *P. cordatum* also had a visible forked-tail, oral sucker, and pharynx (Fig. 2J–L).

The species of isolated trematodes were identified by amplification of the ITS2 region and verified through BLAST. BLAST analysis of sequences from sequenced trematode larvae showed the highest levels of sequence identity (99% to 100% sequence identity) to referenced sequences in GenBank. We identified five different species of trematodes, including *Australapatemon* sp., *Echinostoma* sp., *E. revolutum*, *H. conoideum*, and *P. cordatum*. The lengths of ITS2 were 292 bp, 429 bp, 430 bp, 432 bp, and 294 bp, respectively. The nucleotide sequences obtained in the present study have been deposited in the GenBank database under the accession numbers KM980466–KM980471 (*Australapatemon* sp.), KJ848453–KJ848455 (*Echinostoma* sp.), KM980474 and KM980476–KM980477 (*E. revolutum*), KJ944311–KJ944313 (*H. conoideum*) and OL870492 (*P. cordatum*).

### Prevalence of trematodes in *O. ollula*

The overall trematode infection rate was 22.1% (1818/8238)*. E. revolutum* was detected in the snails from 11 sampling sites, with an infection rate of 13.0% (1069/8238). *Hypoderaum conoideum* infection was detected in the snails from two sampling sites, with an average infection rate of 3.8% (315/8238). *Australapatemon* sp. was detected in the snails from 2 sampling sites, with an infection rate of 2.5% (206/8238). Infection of *P. cordatum* and *Echinostoma* sp. was detected at one sampling site with infection rates of 0.4% (34/8238) and 2.4% (194/8238), respectively. The overall prevalence and trematodes constituent ratio varied in type 1 and type 2 areas (*P* < 0.01).

### Phylogenetic analyses

**Fig. 3:**
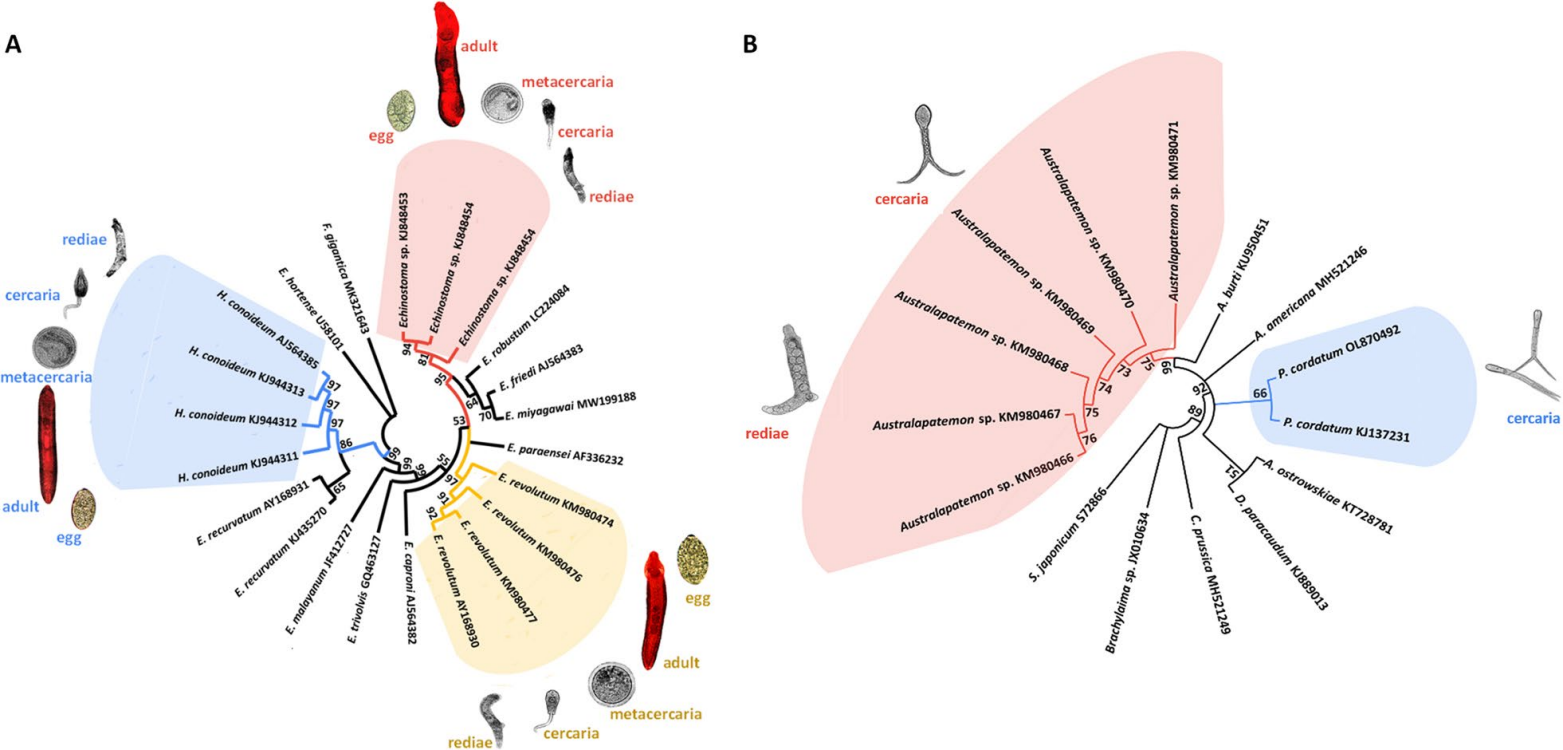
Phylogenetic analyses of isolated trematodes based on the ITS2 sequences and relevant GenBank sequences. **A** Neighbor joining bootstrap consensus tree with 1000 bootstrap iterations for the rediae of echinostomes; **B** Neighbor joining bootstrap consensus tree with 1000 bootstrap iterations for *Australapatemon* sp. and *Pharyngostomum cordatum*

In total, 15 representative high-quality ITS2 sequence data were obtained. Figure 3 shows an NJ tree based on the submitted sequences and relevant GenBank sequences. The ITS2 sequences of *Echinostoma* sp. constituted a monophyletic clade (Fig. 3A shaded pink area), distinct from the clade formed by *E. robustum*, *E. friedi*, and *E. miyagawai*. The sequences of *E. revolutum* and *H. conoideum* constituted a monophyletic group together with *E. revolutum* (AY168930) and *H. conoideum* (AJ564385) references (Fig. 3A shaded blue area). The ITS2 sequences of *Australapatemon* sp. formed a group with *A. burti* (KU950451) at a 99% bootstrap value but formed a unique clade at 75% bootstrap value. Figure 3B shows that the ITS2 sequences of *P. cordatum* were identical to the reference sequences of *P. cordatum* (KJ137231).

### Laboratory infection experiment with *Echinostoma* sp., *E. revolutum*, and *H. conoideum*

We conducted an infection experiment for three kinds of trematode to evaluate the rates of parasite establishment in ducklings. Ducklings were individually exposed to *Echinostoma* sp. (*n* = 52), *E. revolutum* (*n* = 53), and *H. conoideum* larvae (*n* = 57), and all were successfully infected. During subsequent observation on ducklings (17 dpi) fed with *Echinostoma* sp. infected *O. ollula*, we detected eggs [(195.8 ± 3.1) × (143.8 ± 1.6) μm] in the feces, and the morphological characteristics of adult *Echinostoma* sp. were presented as measurements: body length (9.8 ± 1.8 mm), width (1.2 ± 2.3 mm), oral sucker [(638.9 ± 1.9) × (399.2 ± 15.8) μm], acetabulum [(1591.2 ± 17.8) × (1338.2 ± 19.6) μm], pharynx [(492.6 ± 54.3) × (331.7 ± 74.8) μm], anterior testis [(1120.5 ± 139.6) × (707.4 ± 49.2) μm], posterior testis [(1274.9 ± 181.1) × (880.4 ± 49.2) μm], and ovary [(818.9 ± 10.2) × (527.9 ± 48.3) μm].

In contrast, we found *H. conoideum* eggs in ducklings fed with infected *O. ollula* from three sites after a median duration of 12 dpi (range: 9 dpi to 14 dpi). The morphological characteristics of adult *H. conoideum* were as follows: body length (1.05 ± 0.61 mm), width (1.5 ± 0.27 mm), oral sucker [(424.2 ± 16.1) × (293 ± 11.2) μm], acetabulum [(1610.2 ± 98.4) × (1594.6 ± 198.7) μm], pharynx [(379.8 ± 53.2) × (253.6 ± 44.9) μm], anterior testis [(1902.6 ± 164.6) × (875.3 ± 140.8) μm], posterior testis [(2045.2 ± 255.8) × (898.2 ± 139.2) μm], ovary [(751.7 ± 129.3) × (553.9 ± 110.6) μm], and the possession of 50 spines. *E. revolutum* eggs [(104.1 ± 15.1) × (63.1 ± 12.6 μm] were found on 10 dpi. The morphology of the adult *E. revolutum* was characterized by: body length (8.5 ± 1.2 mm), width (2.2 ± 0.7 mm), oral sucker [(260.0 ± 32.5) × (180.1 ± 19.8) μm], acetabulum [(741.6 ± 23.7) × (598.3 ± 25.1) μm], pharynx [(193.1 ± 30.5) × (150.6 ± 27.8) μm], anterior testis [(628.0 ± 31.2) × (459.4 ± 27.8) μm], posterior testis (725.5 ± 17.1 × 557.9 ± 18.7 μm), ovary [(411.8 ± 18.5) × (311.2 ± 15.4) μm], and the presence of a head collar with 37 spines.

### Developmental characteristics of *E. revolutum*, from juvenile to adult, in duckling hosts

**Fig. 4:**
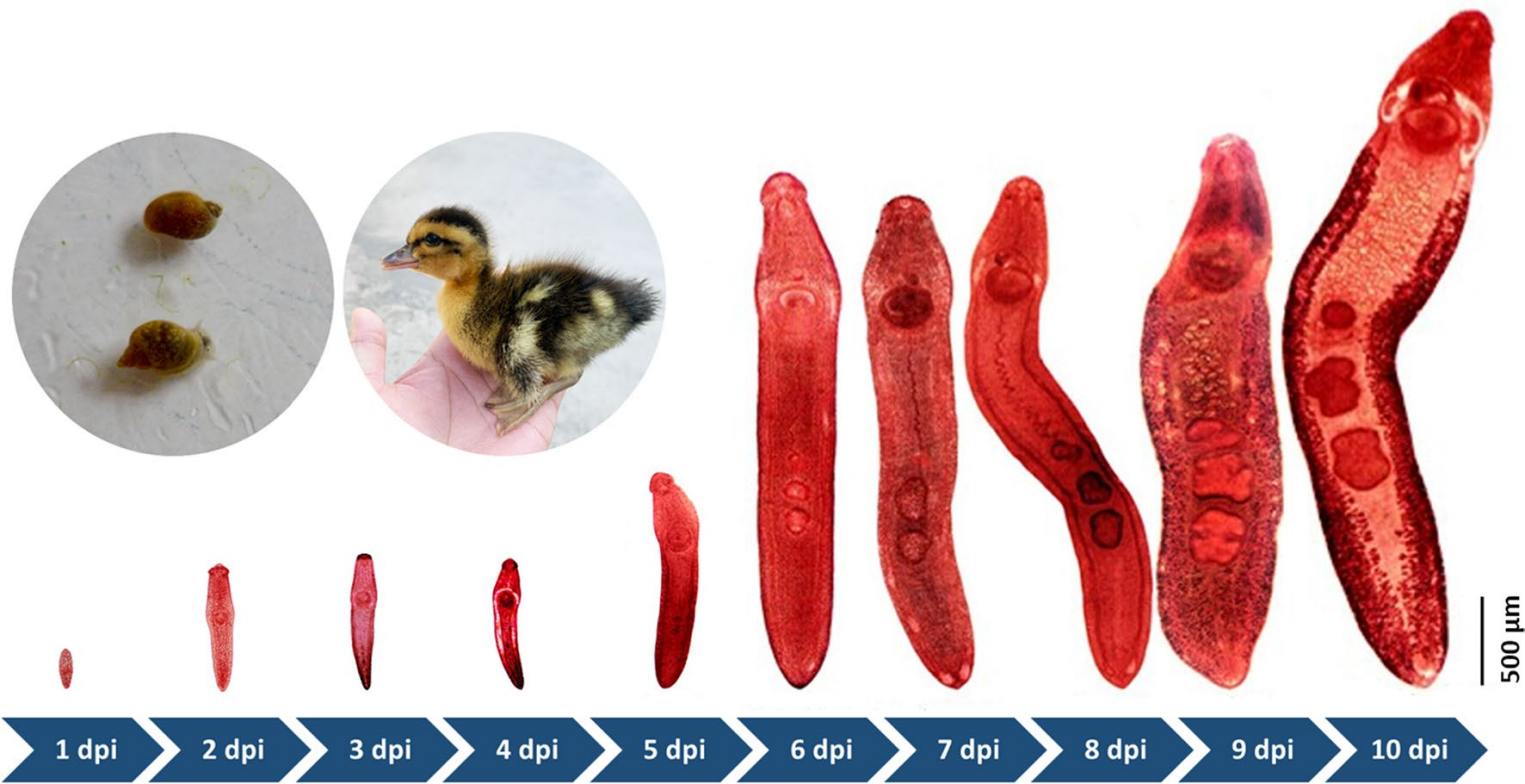
Development of *E. revolutum* in duckling host from 1 to 10 dpi

Experiments were only designed to study the development of *E. revolutum* in duckling hosts, because there were insufficient snails infected with metacercariae of *Echinostoma* sp. and *H. conoideum*. The developmental characteristics of *E. revolutum* were recorded by dissecting infected ducklings from 1 to 10 dpi when eggs in the feces were first detected. *E. revolutum* could be obtained in the small intestine from 1 to 7 dpi and then migrate and reside in the cecum and colon from 8 to 10 dpi. The body length developed from 490 μm to 8500.5 μm (a 17-fold increase). At 1 dpi, juveniles had a circumoral collar bearing 37 spines in a double circle and characterized by clearly visible oral suckers, acetabulum, pharynx, esophagus, and cecum. At 1 dpi, the tiny structure of the testis appeared. By 4 dpi, the ovaries were beginning to organize and develop, and the seminal receptacle began to form. The tubular-shaped uterus loomed at 4 dpi, and maturation of the reproductive and digestive organs occurred at 6 to 8 dpi. The vitelline glands were the last to appear, and several eggs deposited in the uterus were observed at 9 dpi. *E. revolutum* larvae matured at 10 dpi and excreted eggs (Fig. 4). The daily development of *E. revolutum* development was recorded in detail, and the results are shown in Additional file 3: Table S2.

## Discussion

Many species of food-borne trematodes are endemic in developing nations and significantly impact public health [21–23]. Primary research has found that the Lymnaeidae is the dominant host snail for transmitting *Fasciola* sp. [24], and subspecies of *Austropeplea*, *Galba*, *Lymnaea*, *Radix*, and *Stagnicola* could act as intermediate hosts of other zoonotic trematodes [11, 13]. The results reported here demonstrated the presence of five trematode species in collected *O. ollula* samples in Guangxi, China. Morphological characteristics combined with molecular markers identified different developmental stages of *E. revolutum*; *H. conoideum*; *Australapatemon* sp., *P. cordatum*, and *Echinostoma* sp. However, our study did not detect the previously reported *Fasciola* in Yunan Province, although Guangxi is an important region of ruminant fascioliasis as formerly documented [25]. It is possible the sites we studied were not fascioliasis endemic areas. Another possible reason is that we only collected *O. ollula* in limited quantity from typical breeding sites.

In contrast, our results showed that *E. revolutum* was the most prevalent trematode species in Guangxi, with an infection rate of 13.0% among the collected snails. The findings highlight these differences in the environment may thus have a possible effect on driving parasite infection status. This is also showcased by varied zoonotic trematodes infection rates in freshwater snails by Wang et al. [26] and Mereta et al. [27]. Wang et al. reported an overall lower infection rate of 2.27% (3/132) from 9 collection sites in Jilin Province, China, compared with our results. Moreover, similar to our results, no coinfection of trematodes was observed. In Mereta’s study, the overall trematode infection was 3.6% in snails collected from southwest Ethiopia, and coinfection of trematodes was found in four different species. Interestingly, they further explored the relationship between cercarial infection and environmental variables and concluded that environmental determinants largely influenced snail species' abundance, occurrence, and infectivity. In our study, we also found trematode prevalence is associated with the type of habitat. Therefore, environmental infection transmission system and other models include meteorological variables, and validated biological data should be introduced to determine the relationship between environmental influences, socio-economic and demographic risk factors for species distribution, and epidemiological trends.

Zoonotic trematodes are tiny parasites that mainly parasitize the intestines of birds, mammals, and humans [24, 28, 29]. However, due to their high species diversity and indistinguishable morphology, many species have not been given precise classification and investigated. Initially, Jousson et al. proposed the application of gene markers or restriction fragment length polymorphism for molecular identification [30]. Soon afterward, mitochondrial and nuclear ribosomal DNA sequences, particularly ITS2 species-specific markers, appeared as a more suitable genetic marker for identifying and differentiating trematode species. Some isolated trematodes larvae from our study appeared morphologically indistinguishable, in particular for cercaria. Assisted by the ITS2 molecular tool, we obtained reliable results in identifying and differentiating trematode taxa consistent with morphological analyses.

Furthermore, the evolutionary relationships of trematode species were successfully elucidated by distinct monophyletic clades compared with paralogs’ reference sequences deposited in public databases. However, we should be cognizant that molecular and morphological techniques have their own merits and demerits. Different approaches should be applied in different scenarios, and an optimized combination of tools may be necessary for access to speciation and molecular systematic.

Animal models are valuable resources for exploring the biology, life cycle, and pathology of trematode infection. Initially, we tried to establish animal models for all isolated trematode species in our study. Unfortunately, only one model for *E. revolutum* infection was successfully established. Echinostomes are common intestinal parasitic trematodes in poultry. They mainly affect the growth and development of poults while they are less harmful to adults. The developmental cycle of echinostomes in their definitive hosts is short and uncomplicated [31]. Therefore, the animal model for echinostomes will help elucidate the interaction between the trematode and its host [32, 33]. Thus, our research results can also be used to reference other small intestinal flukes that induce an immune response in the definitive host. The present study mainly focused on observations of the growth and development of echinostomes from decapsulation of the cyst to the sexually mature adult stage in the intestinal tract of the host. For the experimental animals, mammals are not susceptible to echinostomes infection, so ducklings were used as the definitive host in this study. Although other animal models have been reported for some echinostomes, the different Echinostomatidae has certain differences in their biology and life cycle [32]. Future animal studies on various aspects of echinostome infection are still needed.

Although we unveiled the zoonotic trematode prevalence in *O. ollula* and established an experimental animal model, there are several limitations in the present study. Firstly, we only investigated zoonotic trematode prevalence in *O. ollula*. Previous studies showed that *Radix plicatula*, *R. swihoei*, and *Gyraulus convexiusculus* are wide distributed snail species in Guangxi and can act as intermediate hosts for trematodes [34]; especially, some species have low intermediate host specificity [35]. These results imply that the actual infection rate of trematodes may be higher than our results reported here. Another limitation of the present study is that we only established one trematode animal model, although we obtained five species during the dissection of collected snails. Usually, it is time-consuming to study the zoonotic trematode life cycle by reintroducing the larva to intermediate and definitive hosts. The isolation of sufficient metacercariae and the selection of appropriate hosts are decisive factors for success. However, there are no guidelines on systematic selection of suitable host for trematode experimental infection. Thus, more candidate animals should be used for screening and evaluating feasibility and stability as hosts.

## Conclusions

The present study revealed the high prevalence of trematodes in *O. ollula* from Guangxi, China. The varied prevalence of trematodes may be associated with the distribution of *O. ollula* and their habitat types. The experimental model of *E. revolutum* revealed the detailed developmental processes in ducks. The study results provide general information and disclose experimental laboratory models for assessing the potential zoonotic echinostomiasis from *O. ollula*. Additional research is needed to clarify the risk of human infection and risk evaluation to ameliorate unwanted adverse effects from exposure to infected *O. ollula*.

## Supplementary Information

**Additional file 1**. Number of snails collected and infected with trematodes in the sampled sites located at Guangxi Autonomous Region.

**Additional file 2: Table S1 **Number of snails collected and infected with trematodes in the sampled sites located at Guangxi Autonomous Region.

**Additional file 3: Table S2**. Development of *E. revolutum* larvae in the duckling host (1 dpi–10 dpi, unit: μm).

## Data Availability

The sequence data have been submitted to GenBank and have been released to the public database before Dec 1, 2021. The GenBank accession numbers are KX781395 for the ITS2 of *Australapatemon* sp., KM980463–KM980465, KM980478–KM980479 for the *Hypoderaeum conoideum*, KM980474 and KM980476–KM980477 for the *Echinostoma revolutum*.
