# Supplementary figures and images for: A relatively high zoonotic trematode prevalence in Orientogalba ollula and the developmental characteristics of isolated trematodes by experimental infection in the animal model
Source: Infect Dis Poverty. 2022 Aug 19;11:91. doi: 10.1186/s40249-022-01014-7 (PMC9389801; doi:10.1186/s40249-022-01014-7)

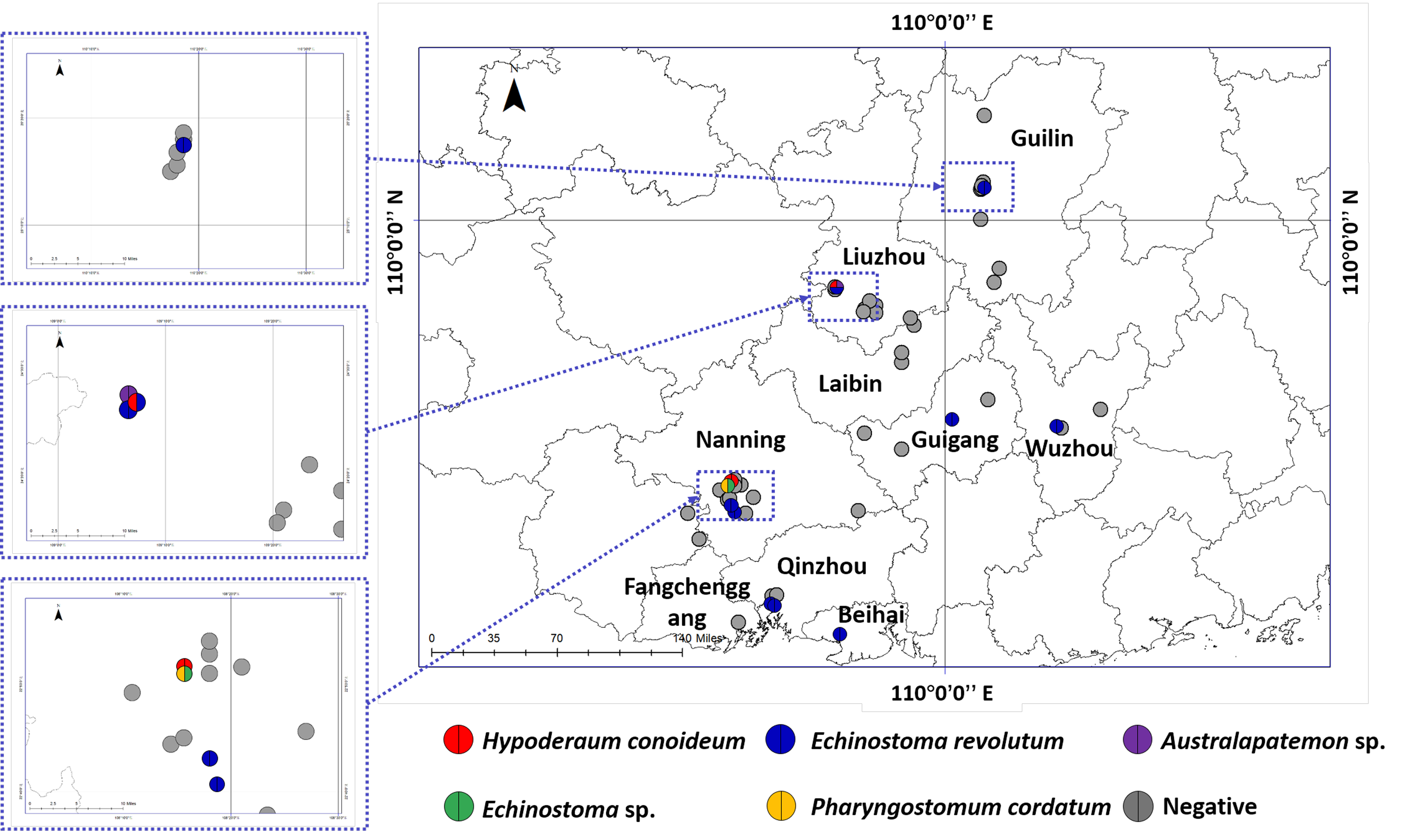

Supplement: Supplementary file 1 — Additional file 1. Number of snails collected and infected with trematodes in the sampled sites located at Guangxi Autonomous Region. [file 40249_2022_1014_MOESM1_ESM.tif]
