# Supplementary material for: A relatively high zoonotic trematode prevalence in Orientogalba ollula and the developmental characteristics of isolated trematodes by experimental infection in the animal model
Source: Infect Dis Poverty. 2022 Aug 19;11:91. doi: 10.1186/s40249-022-01014-7 (PMC9389801; doi:10.1186/s40249-022-01014-7)
Supplement: Supplementary file 2 — Additional file 2: Table S1 Number of snails collected and infected with trematodes in the sampled sites located at Guangxi Autonomous Region. [file 40249_2022_1014_MOESM2_ESM.docx]

Table S1 Number of snails collected and infected with trematodes in the sampled sites located at Guangxi Autonomous Region

| Site | Types of area | Latitude | Longitude | Date | Species | No. of positive/No. of examined (%) |
| --- | --- | --- | --- | --- | --- | --- |
| **Nanning City** | | | | | | |
| Qutou village | 1 | 22.42’N | 108.01’E | 2012.3.17 | - | 0/187 (0.0) |
| Shibu town | 1 | 22.82’N | 108.18’E | 2012.4.14 | - | 0/201 (0.0) |
| Longgang village | 1 | 22.76’N | 108.45’E | 2016.6.22 | - | 0/153 (0.0) |
| Xinlian village | 1 | 22.68’N | 108.31’E | 2016.2.22 | *Echinostoma revolutum* | 107/149 (71.8) |
| Luo village | 1 | 22.74’N | 108.24’E | 2017.7.9 | - | 0/98 (0.0) |
| Heng county | 1 | 22.65’N | 109.30’E | 2018.11.9 | - | 0/167 (0.0) |
| Ji village | 1 | 22.86’N | 108.35’E | 2014.8.16 | - | 0/58 (0.0) |
| Nama town | 1 | 22.63’N | 108.39’E | 2017.7.8 | - | 0/156 (0.0) |
| Fusui town | 1 | 22.63’N | 107.92’E | 2017.3.17 | - | 0/178 (0.0) |
| Luxian mountain | 1 | 22.88’N | 108.30’E | 2018.4.27 | - | 0/200 (0.0) |
| Si village | 1 | 22.90’N | 108.30’E | 2018.7.5 | - | 0/133 (0.0) |
| Liu village | 1 | 22.75’N | 108.26’E | 2019.7.9 | - | 0/147 (0.0) |
| Xiuxaing village | 1 | 22.85’N | 108.30’E | 2020.8.4 | - | 0/68 (0.0) |
| Tianbao reservoir | 1 | 22.85’N | 108.26’E | 2013.5.11 | *Pharyngostomum cordatum* | 34/112 (30.4) |
|  | 1 | 22.85’N | 108.26’E | 2019.8.16 | *Echinostoma* sp. | 57/166 (34.3) |
|  | 1 | 22.85’N | 108.26’E | 2020.8.29 | *Echinostoma* sp. | 137/200 (68.5) |
| Hede village | 2 | 22.86’N | 108.26’E | 2017.4.15 | *Hypoderaum conoideum* | 28/132 (21.2) |
| Naliang village | 2 | 22.72’N | 108.30’E | 2017.6.22 | *E. revolutum* | 64/177 (36.2) |
| **Liuzhou City** | | | | | | |
| Yujia village | 1 | 24.28’N | 109.35’E | 2018.10.3 | *-* | 0/124 (0.0) |
| Shuinan village | 1 | 24.31’N | 109.44’E | 2018.10.4 | - | 0/145 (0.0) |
| Yunjiang town | 1 | 24.15’N | 109.75’E | 2015.11.7 | - | 0/155 (0.0) |
| Shilong town | 1 | 23.28’N | 109.35’E | 2015.11.8 | - | 0/171 (0.0) |
| Liunan district | 1 | 24.25’N | 109.44’E | 2019.11.20 | - | 0/184 (0.0) |
| Lutang village | 1 | 24.46’N | 109.11’E | 2014.4.8 | *Australapatemon* sp. | 77/98 (78.5) |
|  | 1 | 24.46’N | 109.11’E | 2015.6.27 | *-* | 0/214 (0.0) |
| Huxi bridge | 1 | 24.35’N | 109.39’E | 2016.10.3 | - | 0/64 (0.0) |
| Daojiang village | 1 | 24.21’N | 109.72’E | 2016.11.7 | - | 0/56 (0.0) |
| Ertang town | 1 | 23.85’N | 109.65’E | 2016.11.7 | - | 0/113 (0.0) |
| Xiangzhou county | 2 | 23.93’N | 109.65’E | 2016.11.8 | - | 0/146 (0.0) |
| Chashan village | 2 | 24.26’N | 109.34’E | 2017.11.21 | - | 0/145 (0.0) |
|  | 2 | 24.45’N | 109.12’E | 2012.4.7 | *H. conoideum* | 49/102 (48.0) |
|  | 2 | 24.45’N | 109.12’E | 2015.5.17 | *E. revolutum* | 61/167 (36.5) |
|  | 2 | 24.45’N | 109.12’E | 2017.6.27 | *H. conoideum* | 38/203 (18.7) |
|  | 2 | 24.45’N | 109.12’E | 2019.8.10 | *E. revolutum* | 163/200 (81.5) |
| Liushan town | 2 | 24.45’N | 109.12’E | 2018.11.22 | *H. conoideum* | 112/142 (78.9) |
|  | 2 | 24.45’N | 109.12’E | 2018.5.17 | *H. conoideum* | 88/214 (41.1) |
| Guangrong village | 2 | 24.44’N | 109.11’E | 2016.11.22 | - | 0/89 (0.0) |
|  | 2 | 24.44’N | 109.11’E | 2017.4.8 | *E. revolutum* | 52/67 (77.6) |
|  | 2 | 24.44’N | 109.11’E | 2020.6.27 | *E. revolutum* | 173/197 (87.8) |
| Liutang town | 2 | 24.46’N | 109.11’E | 2017.4.8 | *Australapatemon* sp. | 129/158 (81.6) |
|  | 2 | 24.46’N | 109.11’E | 2016.6.27 | - | 0/112 (0.0) |
| **Guilin City** | | | | | | |
| Yanshan town | 1 | 25.85’N | 110.32’E | 2017.10.1 | - | 0/78 (0.0) |
| Xiao village | 1 | 25.30’N | 110.31’E | 2018.5.10 | - | 0/47(0.0) |
| Liushizhou | 1 | 25.31’N | 110.31’E | 2020.5.10 | - | 0/66 (0.0) |
| Yangming town | 1 | 25.01’N | 110.29’E | 2019.5.11 | - | 0/153 (0.0) |
| Malin town | 1 | 24.61’N | 110.44’E | 2018.5.24 | - | 0/38 (0.0) |
| Baiguoshu village | 1 | 25.25’N | 110.29’E | 2016.10.2 | *-* | 0/71 (0.0) |
| Xiaguan village | 1 | 25.26’N | 110.30’E | 2018.5.10 | *-* | 0/135 (0.0) |
| Liutang town | 1 | 25.01’N | 110.29’E | 2019.5.11 | *-* | 0/86 (0.0) |
| Licheng town | 1 | 24.50’N | 110.40’E | 2019.5.24 | *-* | 0/92 (0.0) |
| Qixing village | 2 | 25.28’N | 110.30’E | 2020.10.2 | - | 0/56 (0.0) |
| Shangguan village | 2 | 25.29’N | 110.31’E | 2012.10.1 | *E. revolutum* | 109/143 (76.2) |
| **Beihai City** | | | | | | |
| Beishan village | 1 | 21.67’N | 109.18’E | 2016.6.14 | *E. revolutum* | 56/150 (37.3) |
| **Qinzhou City** | | | | | | |
| Yangwu village | 1 | 21.95’N | 108.61’E | 2016.11.2 | *-* | 0/77 (0.0) |
| Tantou village | 1 | 21.96’N | 108.60’E | 2016.11.2 | *-* | 0/101 (0.0) |
| Xiale village | 1 | 21.97’N | 108.64’E | 2016.11.2 | - | 0/100 (0.0) |
| **Fangchenggang City** | | | | |  |  |
| Tanwu village | 1 | 21.92’N | 108.58’E | 2012.11.1 | *E. revolutum* | 82/133 (61.7) |
| Jianshan village | 1 | 21.93’N | 108.60’E | 2017.11.1 | *E. revolutum* | 75/96 (78.1) |
| Huangzhutang | 1 | 21.75’N | 108.33’E | 2017.11.3 | - | 0/78 (0.0) |
| **Guigang City** | | | | | | |
| Guigang urban | 1 | 23.15’N | 109.65’E | 2018.11.8 | - | 0/67 (0.0) |
| Pingnan county | 1 | 23.55’N | 110.35’E | 2018.11.8 | - | 0/104 (0.0) |
| Xintang village | 1 | 23.38’N | 110.05’E | 2018.11.9 | *E. revolutum* | 63/77(81.8) |
| **Wuzhou City** | | | | | | |
| Wuzhou urban | 1 | 23.47’N | 111.26’E | 2012.11.19 | - | 0/89 (0.0) |
| Teng village | 1 | 23.35’N | 110.91’E | 2012.11.19 | *E. revolutum* | 64/110 (58.2) |
| Cangwu village | 1 | 23.32’N | 110.94’E | 2017.11.19 | - | 0/143 (0.0) |
