# Supplementary material for: A relatively high zoonotic trematode prevalence in Orientogalba ollula and the developmental characteristics of isolated trematodes by experimental infection in the animal model
Source: Infect Dis Poverty. 2022 Aug 19;11:91. doi: 10.1186/s40249-022-01014-7 (PMC9389801; doi:10.1186/s40249-022-01014-7)
Supplement: Supplementary file 3 — Additional file 3: Table S2. Development of E. revolutum larvae in the duckling host (1 dpi–10 dpi, unit: μm). [file 40249_2022_1014_MOESM3_ESM.docx]

**Table S2. Development of *E. revolutum* larvae in the duckling host (1 dpi–10 dpi, unit: μm)**

| **DPI** | **1** | **2** | **3** | **4** | **5** | **6** | **7** | **8** | **9** | **10** |
| --- | --- | --- | --- | --- | --- | --- | --- | --- | --- | --- |
| **Body length** | 490 | 1417.5 | 1520.3 | 1640.2 | 2548.8 | 2992.2 | 4225.6 | 6100.1 | 7436.6 | 8500.5 |
| **Body width** | 220 | 315 | 320 | 364 | 541.6 | 681.5. | 681.5. | 800.8 | 980.2 | 2200.4 |
| **Collar** | 315 | 93.2×  35.7 | 138.8×  61.1 | 187.3×  118.1 | 299.8×  139.4 | 311.5×  147.5 | 316.2×  154.9 | 353×  185.6 | 505.1×  234.7 | 572×  281.8 |
| **Spines** | 8.9×  2.8 | 12.2×  3.6 | 18.2×  5.8 | 23×  5.8 | 31.4×  7.6 | 37.8×  7.6 | 50.4×  12.3 | 55.2×  12.8 | 62.5×  13.2 | 84.4×  17.6 |
| **Oral sucker** | 33.9×  16.2 | 42.4×  20.1 | 51.6×  32.5 | 55.2×  39.5 | 96.4×  63.6 | 124.7×  90.2 | 138.7×  92.9 | 149.6×  98.4 | 197.5×  121 | 260×  180.1 |
| **Acetabulum** | 120.3  ×115.4 | 149.6×  154.5 | 175.3×  152.4 | 182.6×  154.2 | 319.4×  302.9 | 393.9×  308.9 | 452.8×  356.4 | 486.3×  439.6 | 559.7×  466.9 | 741.6×  598.3 |
| **Prepharynx** | 23.1×  12.3 | 31.8×  20.4 | 45.6×  27.2 | 65.3×  60.5 | 102.8×  86.6 | 117.8×  95.7 | 125.4×  85.3 | 124.8×  94 | 136.7×  140.4 | 193.1×  150.6 |
| **Esophagus** | 87.2 | 116.5 | 140.1 | 156.1 | 241.2 | 278.4 | 274.3 | 308.2 | 323.5 | 388.2 |
| **Anterior testis** | — | — | 20.8×  19.7 | 32.1×  21.7 | 79.9×  54.5 | 217.7×  122.5 | 318.7×  249.6 | 379.2×  315.7 | 505.1×  381.2 | 628×  459.4 |
| **Posterior testis** | — | — | 26.3×  25.9 | 38.6×  29.9 | 96.3×  63.1 | 206.4×  143.1 | 357×  258.4 | 447.5×  356.7 | 501.1×  362 | 725.5×  557.9 |
| **Ovary** | — | — | — | 12.3×  14.5 | 70×  42.9 | 90.5×  65.5 | 127×  108.1 | 212.9×  161.7 | 292.3×  191.8 | 411.8×  311.2 |
